# Changes in the Concentration of Purine and Pyridine as a Response to Single Whole-Body Cryostimulation

**DOI:** 10.3389/fphys.2021.634816

**Published:** 2021-01-27

**Authors:** Wioleta Dudzinska, Anna Lubkowska

**Affiliations:** ^1^Institute of Biology, University of Szczecin, Szczecin, Poland; ^2^Department of Functional Diagnostics and Physical Medicine, Pomeranian Medical University in Szczecin, Szczecin, Poland

**Keywords:** cryostimulation, energy metabolism, purines, pyridine, erythrocytes

## Abstract

To our knowledge, this is the first study in which we provide evidence that a single whole-body cryostimulation treatment leads to changes associated with erythrocyte energy metabolism. These changes are beneficial from the point of view of cellular bioenergetics, because they are associated with an increase in ATP concentration and erythrocyte energy potential expressed by an increase in the ATP/ADP and ATP/AMP ratios and the value of adenylate energy charge (AEC). In addition, as affected by cryogenic temperatures, there is a decrease in the concentration of purine catabolism products, i.e., inosine and hypoxanthine in the blood.

## Introduction

Whole-body cryotherapy (cryostimulation) is one of the methods of physical medicine, the therapeutic effect of which is based on the induction and use of the body’s physiological responses to cold. In response to extremely low temperatures (below −100°C), that act for a short time (1–3 min) on the entire surface of the patient’s body, reactions aimed at maintaining thermal homeostasis are triggered (Guillot et al., 2014).

One of the mechanisms being activated under the influence of a strong thermal stimulus in response to the rapid cooling of the skin and subcutaneous tissues (Stocks et al., 2004) is the contraction of surface blood vessels and the redistribution of blood to deeper tissues and organs (centralization of blood circulation). At the same time, α-motoneurons are stimulated via the thermoregulatory center, the tension and metabolic activity of skeletal muscles increase, and consequently the muscles generate significant amounts of heat by shivering thermogenesis. This form of heat production requires a significant amount of energy, which is associated with the need to mobilize energy reserves (muscle glycogen). Shortly after exposure, cutaneous circulation is restored and the blood supply to internal organs increases significantly (Roszkowska et al., 2018). This phenomenon is described as ischemia followed by reperfusion. Such active hyperemia of the whole body, lasting up to several hours after the treatment, intensifies metabolism and oxygen uptake, and facilitates the elimination of harmful metabolism products (Castellani and Young, 2016).

In all living cells metabolic reactions are coupled with each other. The energy released during catabolic processes has to be immediately delivered to cell structures, in which anabolic processes take place. High-energy compounds act as energy carriers in cells, among which adenine nucleotides (ATP, ADP, and AMP) not only couple metabolic reactions with bioenergetic processes, but also work as allosteric control of many regulatory enzymes, so that changes in ATP, ADP, and AMP concentrations can regulate the activity of the entire multi-enzymatic cell network. Therefore, favorable/unfavorable metabolic changes are often a consequence of favorable/unfavorable changes in cellular energetics.

Knowledge about the effects of low temperatures on energy metabolism comes from many studies. It was shown that in mesophilic and thermophilic species, together with temperature increase there is an increase of the level of adenylates, whilst cold or heat shock results in a rapid loss of the concentration of ATP (Napolitano and Shain, 2005).

By contrast, in psychrophilic species the increase of temperature leads to a decrease of adenylate level, whilst heat shock leads to a dramatic decrease of ATP concentration and cold shock to an increase (Napolitano and Shain, 2004; Amato and Christner, 2009).

Research has proven that, ATP concentration of cells in numerous living organisms: bacteria, fungi, algae, plant tissue, plant and human cell lines shows similar changes in response to freezing and thawing. Treatment with cryoprotectant and cooling to up to −196°C led to a significant decrease of ATP content in cells of most model organisms. At the same time, thawing and a longer period of regeneration not only restored ATP concentration to its initial levels, but also often caused it to exceed the initial ones (Bajerski et al., 2018).

Evidence of the effects of low temperatures on energy metabolism also comes from animal studies. It has been shown that exposure of rats to 4°C for 14 consecutive days leads to a significant increase in ATP concentration in tissues with outstanding oxygen metabolism, which, according to the authors, is the body’s protective response to cold stress (Wang et al., 2015). It is also known that metabolic changes observed during hypothermia are a consequence of disturbances in the body’s energy management. With the existing negative energy balance, they consist in reducing the consumption and production of energy in the form of ATP (Erecinska et al., 2003; Froehler and Geocadin, 2007).

Thus, the research results available in the literature show that cold exposure may cause changes in cellular bioenergetics. One of the commonly used treatments in rehabilitation, post training recovery and biological regeneration, during which the whole body in exposed to extremely low temperatures in short time periods (1–3 min) is whole body cryostimulation.

Therefore, the hypothesis of this study is based on the question of whether the stimulatory effect of extremely low temperatures leads to changes in cellular energy metabolism, which is most often assessed by measuring the concentration of purine nucleotides and their metabolites. Hence, our research was based on the assessment of their concentration in red blood cells and plasma.

To date, purine nucleotide transformations and erythrocyte energy metabolism in response to whole-body cryostimulation have not been analyzed. Therefore, the main goal of our research was to determine the effect of extremely low temperatures – systemically affecting the concentration of adenine (ATP, ADP, and AMP), inosine (IMP) and pyridine (NAD and NADP) nucleotides. The total concentration of adenine nucleotides in the adenylate pool (TAN) was also analyzed, as well as the ratio of phosphorylated adenine nucleotide concentrations (AEC).

In addition, we determined the effect of cryogenic temperatures on the plasma concentration of purine catabolism products, i.e., inosine, hypoxanthine, xanthine, and uric acid.

## Materials and Methods

### Subjects

Thirty men, aged 31.9 ± 3.2 years, who had never been subjected to any form of cryostimulation, took part in the research. All the subjects were healthy and normotensive, with a body mass index (BMI) between 22.05 and 28.98 (Table 1). They were all volunteers and were fully aware of all the procedures and the design of the research. They were fully informed about any possible risks and discomfort associated with the experimental procedure, as well as their rights according to the Declaration of Helsinki. All participants agreed to take part and filled the written informed consent and health history questionnaire. The local Ethics Committee (Ethics Committee of Pomeranian Medical University; Ref. KB-0012/54/10) fully approved the study. Also, one of the requirements before the experiment could take place was that all subjects participated in initial medical qualifications, so that the ones will potential contraindications to whole-body cryostimulation could be eliminated. Information about body height, body mass was collected and BMI was calculated as weight (in kilograms) divided by height (in meters) squared (Table 1).

**TABLE 1:** A characteristics of the study group.

	Means ± (SD)	Min	Max
Age	31.9 ± 3.2	25	37
Body height (cm)	177.65 ± 4.9	163	186
Body mass (kg)	81.1 ± 6.2	66	92
BMI (kg/m^2^)	25.7 ± 1.8	22	29
SBP (mmHg)	128 ± 5	102	132
DBP (mmHg)	76 ± 8	63	83
HR_rest_ (bpm)	72 ± 9	64	86

*BMI, body mass index; SBP, systolic blood pressure; DBP, diastolic blood pressure; HR, heart rate.*

### Cryostimulation Procedure

Cryostimulation sessions took place between 9 a.m. and 10 a.m. The participants were non-smokers and were asked not to drink coffee, tea, or cola drinks during the days of the experiment. They were exposed once to a 3 min session of extremely low temperature (−130°C) in a two-stage cryogenic chamber in a group of maximum two participants.

Directly before the treatment the participants’ systolic blood pressure (SBP), diastolic blood pressure (DBP), and heart rate (HR) were measured by a clinically validated automatic blood pressure monitor (OMRON). Glasses, contact lenses and all items of jewelry were removed before entry to the chamber. The body was dried thoroughly to eliminate the sensation of cold and noses and mouths were secured with a surgical mask. An approximately 30 s period in the vestibule at a temperature of −60°C preceded the entry to the cryochamber. During the cryostimulation treatment, the participants were dressed only in shorts, socks, wooden clogs, gloves, and a hat covering the auricles against frostbite. The participants were advised to avoid holding their breaths while in the cryochamber.

### Sample Collection

Blood samples were obtained from an antecubital forearm vein after a 10 min rest in a sitting position; Vacutainer System tubes with EDTA-K_2_ were used (Sarstedt, Germany). Blood samples were taken after overnight fasting, on an empty stomach in the morning between 08:00 and 08:30 (sample T0), 30 min after cryostimulation (sample T30) and the next morning after an overnight fasting at about the same time as previously (sample T24).

The whole blood was centrifuged (Universal 320R, Hettich Lab Technology, Tuttlingen, Germany) within 3 min after drawing a sample (1,000 × *g*, 5 min, 4°C). Plasma and buffy coat were removed. Then, plasma was divided into aliquots and immediately deep-frozen at −80°C until the analysis. Erythrocytes were washed with buffered 0.9% NaCl solution and centrifuged (1,000 × *g*, 5 min, 4°C). The nucleotide concentration was determined in washed erythrocytes and expressed in relation to the volume of erythrocytes as previously described (Pospieszna et al., 2019; Rodziewicz et al., 2020).

### Determination of Purines and Pyridines

The purine nucleotide (ATP, ADP, AMP, and IMP) and pyridine nucleotide (NAD and NADP) concentrations were determined in erythrocytes. The inosine, hypoxanthine, xanthine, and uric acid concentrations were determined in the plasma. The concentrations of purine and pyridine, which were analyzed, were determined by high-performance liquid chromatography (HPLC). It was done in accordance with the method described in detail by Smolenski et al. (1990) and used by us (Dudzinska et al., 2018; Pospieszna et al., 2019).

### Erythrocyte Purine and Pyridine Nucleotides

The samples of washed erythrocytes (500 μl) were deproteinised with an equal volume of 1.3 mol/l perchloric acid (HClO_4_), mixed, and then centrifuged at 12,000 rpm for 5 min at 4°C. 600 μl of supernatant was neutralized with 130–160 μl of 1 mol/l K_3_PO_4_ (to pH 5–7). The samples were centrifuged again under the same conditions as previously, and supernatant was stored at −80°C until future analysis.

The concentrations of purine (ATP, ADP, AMP, and IMP) and pyridine (NAD and NADP) nucleotides being determined were expressed in relation to erythrocyte volume. The intra-erythrocyte concentrations of purine and pyridine nucleotides are expressed as μmol/l RBC. The values of total adenine nucleotide pool (TAN = [ATP] + [ADP] + [AMP]) and AEC were also calculated $\left(\text{AEC}=\frac{\left[\mathrm{ATP}\right]+0.5\,\left[\mathrm{ADP}\right]}{\left[\mathrm{ATP}\right]+\left[\mathrm{ADP}\right]+\left[\mathrm{AMP}\right]}\right)$

### Plasma Inosine, Hypoxanthine, Xanthine, and Uric Acid

Plasma was later deproteinised with 1.3 mol/l HClO_4_ and once more centrifuged (14,000 rpm, 5 min, 4°C). An acid supernatant was neutralized with 1 mol/l K_3_PO_4_ (to pH 5–7), centrifuged (14,000 rpm, 5 min, 4°C), and stored at −80°C before analysis.

The concentrations of inosine, hypoxanthine, xanthine, and uric acid were determined in the plasma and expressed as μmol/l of plasma.

### Chromatographic Conditions

We separated the purine nucleotides: ATP, ADP, AMP, and IMP; pyridine nucleotides: NAD, NADP; purine metabolism products: inosine, hypoxanthine, xanthine, and uric acid with the HPLC method of Smolenski et al. (1990). Sample aliquots (100 μl) were injected into the chromatography column and the nucleotides were separated using a linear phosphate buffer gradient system (buffer A: 150 mmol KH_2_PO_4_, 150 mmol KCl adjusted to pH 6.0 with K_2_HPO_4_; buffer B: 15% v/v solution of acetonitrile in buffer A) at a flow rate at 0.666 ml/min. The peaks were detected by absorption measurements at 254 nm. The composition of the mobile phase was controlled by a low-pressure gradient mixing device. The cycle time was 12.8 min between injections. The analytical column was maintained at a constant temperature of 20.5°C.

## Statistical Analysis

Statistical analysis was performed using one-way analysis of variance (ANOVA) with *post hoc* comparisons (Tukey’s HSD test). The normality of the distribution was confirmed by the Kolmogorov-Smirnov test, while the homogeneity of variances was checked by Levene’s test. The level of statistical significance *p* < 0.05 was considered statistically significant. The results are presented as mean values (±standard deviation).

## Results

### Adenine Nucleotides

**FIGURE 1:**
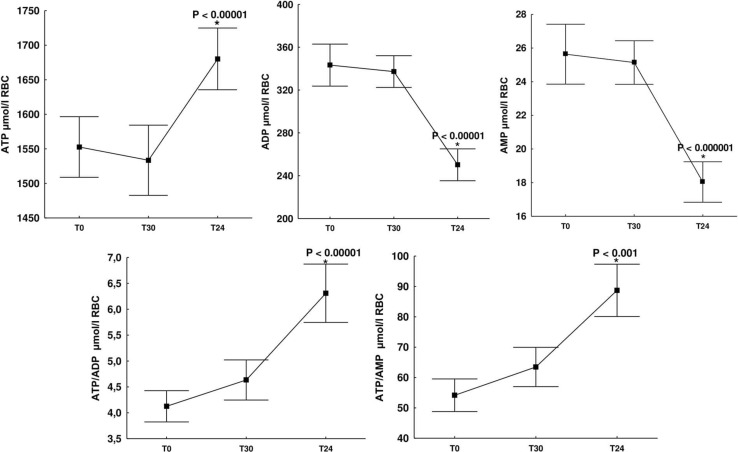
Change in adenine nucleotide concentrations (ATP, ADP, and AMP), ATP/ADP, ATP/AMP ratios in erythrocytes of healthy subjects at rest (T0), 30 min after cryostimulation (T30), in the morning the day after (T24). ATP, adenosine-5′-triphosphate; ADP, adenosine-5′-diphosphate; AMP, adenosine-5′-monophosphate. Values are given as means ± SD. *Significant difference between rest (T0) and 30 min after cryostimulation (T30).

When assessing the effect of a single whole-body cryostimulation treatment on adenine nucleotide concentration, we observed an approximately 10% increase in ATP concentration 24 h after cryostimulation (T24) (1,680 ± 117 μmol/l RBC) compared to concentrations before it (T0) (1,552 ± 115 μmol/l RBC) and 30 min after cryostimulation (T30) (1,533 ± 133 μmol/l RBC). This difference was statistically significant (*p* ≤ 0.00001). At the same time, intra-erythrocyte concentrations of ADP and AMP decreased by approximately 30%, reaching (T24) 250 ± 39 μmol/l RBC and (T24) 18 ± 3.15 μmol/l RBC, respectively. In the case of ADP, this difference was statistically significant (*p* ≤ 0.00001) both in relation to the concentrations before cryostimulation (T0 vs. T24) (343 ± 51 μmol/l RBC vs. 250 ± 39 μmol/l RBC; *p* ≤ 0.00001) and 30 min after it (T30 vs. T24) (337 ± 39 μmol/l RBC vs. 250 ± 39 μmol/l RBC; *p* ≤ 0.00001). Similarly, in the case of AMP, a significant decrease (*p* ≤ 0.000001) was observed both in relation to the values assessed before cryostimulation (T0 vs. T24) (25 ± 4.68 μmol/l RBC vs. 18 ± 3.15 μmol/l RBC) and 30 min after (T30 vs. T24) (24 ± 3.48 μmol/l RBC vs. 18 ± 3.15 μmol/l RBC) (Figure 1).

Despite the fact that extremely low temperatures led to significant changes in ATP, ADP and AMP concentrations, and these changes took place within the TAN pool being the sum of these nucleotide concentrations, it remained comparably relatively constant (Table 2).

**TABLE 2:** Total adenine nucleotide pool, adenylate energy charge and concentration of inosine and pyridine nucleotides in red blood cells before (T0); 30 min after (T30); 24 h after cryostimulation (T24).

	T0	T30	T24
TAN (μmol/l RBC)	1921 ± 134	1895 ± 155	1948 ± 140
AEC	0.89 ± 0.01	0.89 ± 0.009	0.92 ± 0.008*
IMP (μmol/l RBC)	11 ± 2.61	10 ± 2.39	10 ± 2.39
NAD (μmol/l RBC)	69 ± 7.24	68 ± 7.13	69 ± 7.02
NADP (μmol/l RBC)	40 ± 6.39	39 ± 5.39	40 ± 5.25

TAN, total adenine nucleotide pool; AEC, adenylate energy charge; IMP, inosine-5′-monophosphate; NAD, nicotinamide adenine dinucleotide; NADP, nicotinamide adenine dinucleotide phosphate. Values are given as means ± SD.*p ≤ 0.000001; difference between rest (T0) and 30 min after cryostimulation (T30).

Undoubtedly, an important indicator of the cellular energy status is not only the level of adenine nucleotides but also their relationship. A statistically significant increase in ATP concentration with a decrease in ADP and AMP concentration resulted in a significant increase in the ATP/ADP ratio (*p* ≤ 0.00001) and the ATP/AMP ratio (*p* ≤ 0.001) (Figure 1). Immediately before cryostimulation (T0), 30 min (T30) and 24 h after it (T24) the ATP/ADP ratio was 4.62 ± 0.78 μmol/l RBC, 4.58 ± 0.53 μmol/l RBC, and 6.84 ± 0.98 μmol/l RBC, respectively, while that of ATP/AMP concentration: (T0) 62 ± 13 μmol/l RBC, (T30) 62 ± 11 μmol/l RBC and (T24) 95 ± 16 μmol/l RBC, respectively (Figure 1). Therefore, the presented results indicate an increase in the rate of AMP and ADP metabolism to ATP, which allows to explain the significant (*p* ≤ 0.000001) increase in the value of AEC observed 24 h after cryostimulation (Table 2).

### Inosine Nucleotide

In our studies, a single whole-body cryostimulation treatment did not lead to changes in intra-erythrocyte IMP concentration (Table 2).

### Pyridine Nucleotides

In response to a single stimulation with cryogenic temperatures, we did not observe changes in the erythrocyte concentration of pyridine nucleotides: NAD and NADP (Table 2). However, we would like to emphasize that the measurement method used by us only allowed to assess the concentration of the total NAD and NADP pool without the possibility of assessing their components, i.e., oxidized and reduced form of NAD (NAD/NADH) and oxidized and reduced form of NADP (NADP/NADPH).

The NAD(H) and NADP(H) redox couples serve as cofactors or/and substrates for many enzymes to maintain cellular redox homeostasis and energy metabolism (Xiao et al., 2018). Therefore, in our opinion, further research is needed to clarify the relationship between the components of the NAD (H) and NADP (H) pools and how these two redox couples jointly regulate the cellular oxido-reductive state and cellular metabolism in response to the systemic effects of cryogenic temperatures.

### Purine Catabolism Products: Inosine, Hypoxanthine, Xanthine, and Uric Acid

Our data set shows that a single stimulation with cryogenic temperatures led to a significant decrease in the concentration of inosine (*p* ≤ 0.0001) and hypoxanthine (*p* ≤ 0.0001) in plasma. Similarly as in the case of significant changes in the concentration of adenine nucleotides, a significant decrease in inosine and hypoxanthine concentration was observed 24 h after cryostimulation (Table 3). As demonstrated by our findings, a single whole-body cryostimulation treatment does not lead to significant changes in xanthine and uric acid concentration (Table 3).

**TABLE 3:** Concentration of inosine, hypoxanthine, xanthine, and uric acid in plasma before (T0); 30 min after (T30); 24 h after cryostimulation (T24).

	T0	T30	T24
Inosine (μmol/l)	0.37 ± 0.18	0.31 ± 0.12	0.20 ± 0.15*
Hypoxanthine (μmol/l)	1.99 ± 1.32	1.92 ± 1.08	1.19 ± 0.72*
Xanthine (μmol/l)	0.26 ± 0.12	1.08 ± 0.13	0.26 ± 0.11
Uric acid (μmol/l)	216 ± 49	217 ± 45	225 ± 42

Values are given as means ± SD.*p ≤ 0.0001; difference between rest (T0) and 30 min after cryostimulation (T30).

## Discussion

To our knowledge, this is the first study in which the effect of cryogenic temperatures on erythrocyte energy metabolism and the concentration of purine catabolism products in the blood was assessed. The study has shown that a single treatment has no significant effect on total adenylate concentration (TAN), while the concentration of individual adenine nucleotides in the TAN pool have changed significantly. At 24 h after cryostimulation, together with a significant increase in ATP, we observed a significant decrease in ADP and AMP, resulting in an increase in ATP/ADP, ATP/AMP (Figure 1) and AEC (Table 2). In addition, our study has shown that at the same time, under the influence of cryogenic temperatures, there is a significant decrease in the concentration of inosine and hypoxanthine in the blood, which are products of purine catabolism, mainly ATP (Table 3).

The most important finding of this study is that whole body exposure to cryogenic temperatures leads to an increase in ATP concentration in erythrocytes. The synthesis of ATP in erythrocytes is primarily the result of effective glycolysis, which is the only source of ATP, and the ATP/ADP ratio and the AEC value are the basic indicators describing the amount of cellular energy resources, mainly ATP. More precisely, the AEC value characterizes the degree of “saturation” of the TAN pool with high energy bonds. AEC can therefore take values from 0 [when (TAN) = (AMP)] to 1 [when (TAN) = (ATP)] (Dudzinska et al., 2006; De la Fuente et al., 2014). In erythrocytes, purine nucleotides are synthesized solely in reutilization reactions (salvage), which aim to include free purine bases and nucleosides in the purine ring of mononucleotides: AMP, IMP, and GMP (Craig and Eakin, 2000; Schuster and Kenanov, 2005). AMP is introduced into the pool of high-energy adenine nucleotides in a reaction involving an adenylate kinase (EC 2.7.4.3; AMP + ATP ↔ 2ADP) (Abrusci et al., 2007), and ATP resynthesis with ADP takes place in substrate phosphorylation reactions on the glycolysis pathway, which is the only source of ATP (Dudzinska et al., 2006).

Normally, the rate of ATP formation in the cells is equal to the rate of ATP utilization, and the intracellular ATP is maintained at a constant level. According to our findings, ATP concentration increases as ADP and AMP decrease. Since TAN was constant in measurement, and ATP and ADP concentration at a given time is determined by the rate of phosphorylation and dephosphorylation reactions, we conclude that the increase in ATP concentration with stoichiometric decrease in ADP and AMP concentration occurs through the phosphorylation reactions of AMP and ADP to ATP. This direction of changes allows to explain the significant increase in ATP/ADP and ATP/AMP ratios and AEC observed at the same time. Observations that the TAN level does not change are therefore not surprising (Table 2). It is well known that the total adenylate pool size (ATP + ADP + AMP) is regulated by negative feed-back with adenylates (mainly AMP) (Hardie and Hawley, 2001; Ataullakhanov and Vitvitsky, 2002).

When the rate of ATP consumption exceeds the rate of its production, the equilibrium of the reaction catalyzed by adenylate kinase is shifted toward ATP synthesis. The increasing AMP levels stimulate AMP deamination to IMP to stabilize the decreasing AEC. Although the stabilizing effect is evident, the price of this stabilization is the reduction of TAN, ATP concentration and AEC (Ataullakhanov and Vitvitsky, 2002). Therefore, it is unlikely that the decrease in AMP concentration observed 24 h after exposure to extremely low temperatures was caused by the removal of AMP from TAN by deamination to IMP. Our data confirm neither an increase in IMP concentration (Table 2) nor a decrease in ATP concentration and TAN (Table 2). Based on these observations, we come to the conclusion that an extremely low, short-term cryogenic stimulus stimulates the increase in ATP production in red blood cells through the phosphorylation of ADP and AMP.

It has previously been shown that exposure of rats to 4°C for 14 consecutive days leads to a significant increase in ATP concentration in hippocampus, cortex, cerebellum, liver, heart, muscle, and brown adipose tissue (Wang et al., 2015). Another study showed that maintaining energy balance during acute cold exposure (−15°C, 4 h) results in an increase in blood glucose concentration (stimulation of glycogenolysis and gluconeogenesis) and activation of the protein kinase B (Akt) pathway, which promotes the production and increase of ATP concentration in rat liver cells (Wang et al., 2013). A sharp increase in ATP concentration was also found in the liver of male C57BL/6 mice after 6 h of cold exposure (4°C) (Yao et al., 2018). Therefore, existing evidence indicates that, in response to cold stress, many cells increase their energy resources. In this study, we first show an increase in ATP concentration in red blood cells in response to a 3-min, short-term exposure to cold. At the same time, it should be noted that the cells will stop producing ATP if their energy demand decreases and, as a consequence, there will be no increase in ATP concentration. In addition, ATP is not kept in abundance or stored in cells as other biomolecules are, but rather its production is adapted to current energy needs. Thus, we conclude that in the case of exposure to extremely low temperatures, an increase in ATP concentration may result from an increase in energy demand, mainly due to an increase in the activity of pathways dependent on its inflow, including many anabolic reactions (Kennedy et al., 1999; Yaniv et al., 2013). Although we are unable to determine the rate of anabolism and/or catabolism from the available data, it is well known that regulatory enzymes of the anabolic pathways, or ATP-utilizing ones, show an increase in activity with an increase in AEC (De la Fuente et al., 2014; Hardie et al., 2016; Hoxhaj et al., 2017). In our study, a 3-min exposure to −130°C significantly improved erythrocyte AEC value from 0.89 to 0.92 (*p* = 0.000001) (Table 2) and ATP from 1,552 to 1,680 μmol/l RBC (*p* = 0.0001) (Figure 1). Furthermore, previous research (Szygula et al., 2014) have shown increased hemolysis of red blood cells after a series of cryostimulation treatments. Such cell elimination, particularly of the aging ones, the most sensitive, with decreased concentration of ATP in comparison to young ones (Sakuta, 1981), should be considered in the discussion on the reason for the increase of ATP concentration in red blood cells.

Our research provides further interesting results. With the increase in ATP concentration in erythrocytes, we also observed a significant decrease in the concentration of inosine and hypoxanthine in the blood (Table 3). It is important to mention that under normal conditions, inosine and hypoxanthine being present in the blood are derived from normal purine catabolism, mainly ATP, of virtually all types of cells and tissues. The importance of inosine and hypoxanthine as ischemic/hypoxic biomarkers has long been recognized (Hellsten et al., 1999; Farthing et al., 2015). Under these conditions, adenine nucleotides are degraded to IMP via AK and AMP deaminase. Although the majority of the formed IMP is reaminated, a fraction is further degraded to inosine and hypoxanthine, which may diffuse across membranes to blood plasma (Molina-Arcas et al., 2009). Hypoxanthine is then metabolized by xanthine oxidase (EC 1.1.3.22) and/or xanthine dehydrogenase (EC 1.1.1.204) to xanthine and uric acid, purines that cannot be recycled. The formation of these metabolites means an irreversible loss of oxypurines that could be used to synthesize ATP in reutilization reactions (Hellsten et al., 1999). Hypoxanthine phosphoribosyl transferase (HPRT, EC 2.4.2.8) catalyzes a rescue pathway in which hypoxanthine can be converted to IMP and then, through a series of intermediary steps, to ATP (Moriwaki et al., 1999). Because *de novo* nucleotide synthesis is energy expensive, many tissues, especially those with high energy requirements, use rescue pathways extensively for purine synthesis (Johnson et al., 2019). There is evidence from marine mammals that regular ischemia/reperfusion cycles (which in our case occurs as a cardiovascular response to cryostimulation) during diving improve purine recycling via the HGPRT-IMP pathway. This explains the presence of lower plasma hypoxanthine concentration, which is probably used as substrates in ATP resynthesis (López-Cruz et al., 2016). Also, in response to frequent episodes of hypoxia (high intensity training), an increase in HGPRT activity in muscles and erythrocytes explains the significantly lower plasma concentration of inosine and hypoxanthine at rest and after intense physical effort (Hellsten-Westing et al., 1993; Dudzinska et al., 2018; Pospieszna et al., 2019). However, if more efficient purine recycling may be one of the possible reasons explaining the decrease in blood concentration of inosine and hypoxanthine, it is still doubtful to what extent this mechanism explains the changes in inosine and hypoxanthine concentrations after the exposure to extremely low temperatures. Indeed, the level of these metabolites in the blood may depend not only on the performance of rescue pathways, but also of catabolic pathways (Nemkov et al., 2018). The reduced rate of AMP deamination, and thus the degradation of IMP, may result in a decrease in plasma inosine and hypoxanthine concentration (Hellsten-Westing et al., 1993). In addition, as is known, under the influence of a strong, short-term thermal stimulus, tissue hyperemia and increased capillary filtration occur. This may promote the elimination of accumulated metabolic products (Stocks et al., 2004). In studies on the effect of whole-body cryostimulation on the oxidant and antioxidant balance in athletes, it has been shown that hyperemia is the cause of rapid removal of lipid peroxidation products and a decrease in their concentration in the blood (Wozniak et al., 2007; Mila-Kierzenkowska et al., 2009). The decrease in inosine and hypoxanthine concentration observed in our study after cryostimulation may be caused by efficient elimination of purine catabolism products, as suggested by Sarver et al. (2017) who observed a decrease in hypoxanthine muscle concentration 2 h after 15-min of local cryotherapy. Active inosine and hypoxanthine uptake into liver cells and further degradation to uric acid cannot be excluded. In our study, however, a single cryostimulation treatment did not lead to changes in the concentrations of xanthine and uric acid in the blood (Table 3), which is consistent with the report by Lubkowska et al. (2012).

Therefore, we postulate that a short-term exposure to extremely low temperatures can significantly reduce the concentration of inosine and hypoxanthine in extracellular fluid by (1) improving purine recycling on the HGPRT-IMP pathway, which may subsequently increase intracellular ATP concentration, (2) decreasing production of inosine and hypoxanthine, or (3) improving their elimination. The explanation of these mechanisms, however, is outside the scope of this study.

To sum up, for the first time, to the best of our knowledge, we present evidence that a single whole-body cryostimulation treatment leads to changes associated with erythrocyte energy metabolism. These changes are beneficial (desirable) from the point of view of cellular bioenergetics, because they are associated with an increase in intra-erythrocyte ATP concentration and erythrocyte energy potential expressed by the ATP/ADP and ATP/AMP ratios and AEC. Moreover, the purine metabolic response to cryogenic temperatures is also associated with a decrease in the concentration of purine catabolism products, i.e., inosine and hypoxanthine, in the blood. Collectively, the increase in ATP concentration in red blood cells, along with the decrease in plasma inosine and hypoxanthine concentration, gives a strong hypothesis for an increase in energy resources also in other cells.

The obtained results encourage to continue research not only to clarify the biochemical mechanisms of these changes, their relationship with changes in systemic metabolism in response to cryogenic temperatures, but also because interventions that support the production of ATP can have many beneficial effects on health (Koopman et al., 2012; Pathak et al., 2013; Johnson et al., 2019). Finally, the potential role of whole-body cryostimulation in targeting red blood cells (and possibly other cells) to increase energy resources may have important therapeutic implications in planning future research.

The limitations of this study should also be discussed. The assessment and interpretation of changes in the concentration of adenine, inosine and pyridine nucleotides, as a result of the systemic effects of cryogenic temperatures, was based on blood sampling 30 min and 24 h after the procedure, the results were related to the baseline values assessed in the morning before exposure to cryogenic temperatures. Such a scheme was based on previous studies confirming changes in adenylate metabolism 30 min after intense physical exercise and due to earlier observations of changes in the concentration of selected blood biochemical parameters after cryostimulation. Treating the obtained results as the first in this subject, one should certainly consider increasing the number of samples in the post-stimulation period with such a strong thermal stimulus.

In order to avoid over-interpretation of the results or erroneous inference, further research is needed to verify the current findings and to explore the mechanisms underlying our observations in more detail. The presented research can serve as a basis for its continuation and establishing the relationship between the observed changes in the concentration of purines and their metabolites (inosine, hypoxanthine) with changes in systemic metabolism in response to cryogenic temperatures, as well as taking into account the temperature range, stimulus duration and individual factors (age, sex, weight, and body composition) that could affect the extent of the changes.

## Data Availability Statement

The raw data supporting the conclusions of this article will be made available by the authors, without undue reservation.

## Ethics Statement

The studies involving human participants were reviewed and approved by the study was approved by the local Ethics Committee (Ethics Committee of Pomeranian Medical University; Ref. KB-0012/54/10). The patients/participants provided their written informed consent to participate in this study.

## Author Contributions

AL performed the research and was involved in acquisition of the data. WD and AL analyzed and interpreted the data and drafted the manuscript. Both authors were involved in the critical revision of the manuscript and approved the final version of the manuscript.

## Conflict of Interest

The authors declare that the research was conducted in the absence of any commercial or financial relationships that could be construed as a potential conflict of interest.
